# Bone marrow plasma cytokine composition indicates acute myeloid leukemia progression and treatment response

**DOI:** 10.3389/fcell.2026.1844017

**Published:** 2026-06-24

**Authors:** Qiuyu Mo, Guoran Xie, Kangle Huang, Weiye Nie, Hong Chen, Jie Yang, Chang Liu, Xiayun Su, Qunqian Ning, Jie Chang, Duanfeng Jiang

**Affiliations:** 1 Department of Oncology, The Fourth Affiliated Hospital of Guangxi Medical University, Liuzhou, Guangxi, China; 2 Department of Hematology, The Fourth Affiliated Hospital of Guangxi Medical University, Liuzhou, Guangxi, China; 3 Department of Hematology, Hunan Provincial People’s Hospital and The First Affiliated Hospital of Hunan Normal University, Changsha, China

**Keywords:** acute myeloid leukemia, bone marrow plasma, cytokine levels, progression, treatment response

## Abstract

**Background:**

The progression of acute myeloid leukemia (AML) is characterized by intricate interactions between leukemia cells and the bone marrow (BM) microenvironment.

**Methods:**

In this study, we conducted a comprehensive analysis of the plasma cytokine composition in BM samples from patients with AML. A total of 75 BM samples were collected from AML patients consecutively recruited, including 41 newly diagnosed (ND‐AML), 19 complete remission (CR) and 15 relapsed/refractory cases (R/R-AML). Using enzyme-linked immunoadsorbent assay, we analyzed BM plasma levels of interleukin (IL)-1 family (IL-1β, IL-1RA and IL-18), IL-2 family (IL-2, IL-4, IL-13, IL-15 and IL-21), IL-12, interferon-gamma (IFN-γ), innate inflammatory and immune regulatory cytokines [IL-6, IL-10, IL-17, tumor necrosis factor (TNF)-α, and transforming growth factor (TGF)-β].

**Results:**

Our study found significant differences in BM plasma inflammatory and immunologic cytokines between ND-AML, CR, and R/R-AML, with notable changes in LDH, ELN risk, IL-12, IL-18, and IL-21 between incomplete remission (ICR) and CR status. These cytokines were at weakened level in the BM microenvironment of R/R-AML. Concentrations of IL-17 and TGF-β in BM plasma elevated at initial diagnosis, declined after CR, and increased again at relapse, indicating the disease activity.

**Conclusion:**

These findings emphasize the dynamic monitoring of BM plasma cytokines during AML progression, and highlight the significance of cytokine composition in guiding treatment and improving patient outcomes.

## 1 Introduction

Acute myeloid leukemia (AML) is a heterogeneous group of hematopoietic malignancies (Bhagwat et al., 2024), and has largely been treated with chemotherapy, targeted therapy, immunotherapy, and hematopoietic stem cell transplantation (HSCT) (Forsberg and Konopleva, 2024). After initial induction chemotherapy, 60–80% of patients can achieve complete remission (CR). Despite emergence of new therapeutic options, the outcomes of patients with relapsed or refractory AML are consistently disappointing with 5-year overall survival rates of ∼10% (DeWolf and Tallman, 2020). The high recurrence rate and poor outcomes of AML remain urgent clinical challenges, therefore new biomarkers to identify the diagnosis, treatment, and prognosis of AML is urgently needed (Jiao et al., 2025). Duration of first CR was a major factor for risk stratification in relapsed AML, and other prognostic factors include age, cytogenetics at diagnosis, certain molecular features such as FMS-like tyrosine kinase 3 (FLT3)-internal tandem duplication (ITD) mutation status (Döhner et al., 2022). The abnormal expression of microRNAs can be considered as biomarkers for diagnosis, prognosis, and treatment in AML patients (Gandomkar et al., 2026). However, these markers are only used at the time of initial diagnosis to assess prognosis, with changes after treatment and their significance remaining insufficiently evaluated (Small et al., 2022). Additionally, laboratory indicators for monitoring the drug resistance and relapse-initiating properties remains an unmet clinical need (Rodríguez-Medina et al., 2024). Overall, there is a pressing need for more accurate, efficient, and faster prognostic markers in clinical practice.

Cytokines play an important role in the regulation of normal and pathologic hematopoiesis. A pro-inflammatory state, described in hematopoietic malignancies, may participate in clonal selection (Ravalet et al., 2025). Abnormalities in the cytokine signaling pathways are characteristic of all forms of leukemia. In leukemic cells, cytokines are exploited to serve critical parts of the tumor program (Simioni and Neri, 2026). Previous study suggest that various cytokines released by the leukemic cells promote the proliferation and progression of AML cells (Karimdadi Sariani et al., 2021). Carey et al. (2017) found that interleukin (IL)-1 family cytokines including IL-1α, IL-1β, IL-3, and granulocyte macrophage colony stimulating factor (GM-CSF), as well as macrophage colony stimulating factor (M-CSF), granulocyte colony-stimulating factor (G-CSF), and tumor necrosis factor (TNF)-α, stimulated the growth of AML cells (Carey et al., 2017). In another study, (Sanchez-Correa et al., 2013) evaluated the levels of cytokines including IL-1β, IL-2, IL-4, IL-5, IL-6, IL-8, IL-10, IL-12p70, IL-17A, TNF-α, and interferon-γ (IFN-γ) in peripheral blood plasma, and found that higher plasma levels of IL-6, IL-10, and TNF-α in AML patients than that of healthy controls. In addition, an increase in IL-8 in patients less than 65 years, and higher levels of IL-4, IL-5 and IL-12p70 only in elderly patients than that of age-matched healthy controls. Moreover, low IL-6 and high IL-10 levels were considered as favorable prognostic factors in AML (Sanchez-Correa et al., 2013). To identify recurrent cytokine patterns according to AML ontogenic subtypes, (Ravalet et al., 2025) quantified the concentration of 49 cytokines in the bone marrow (BM) plasma from AML, myelodysplastic syndrome (MDS), and healthy volunteers. They confirmed a pro-inflammatory profile with increased concentrations of chemokine ligand (CXCL) 8, CXCL10 and IL-6 when comparing AML to MDS, a pre-AML disease status (Ravalet et al., 2025). These findings support the idea that the abnormalities of cytokines may represent a useful marker for the prediction of clinical evolution in patients with AML.

In this study, we addressed the question of cytokine concentrations in the BM plasma of AML patients with different disease status comprising newly diagnosed AML (ND-AML), CR and relapsed/refractory AML (R/R-AML), as well as different treatment response including CR and incomplete remission (ICR). We particularly explored the association of alterations in these cytokine concentrations with the clinical characteristics, disease status and treatment response. A better understanding of this scenario may be crucial to the identification of AML patients at diagnosis who are at high risk of poor treatment response or outcome to improve their clinical outcomes and to identify biomarkers for early therapeutic intervention.

## 2 Methods

### 2.1 Patients and samples

A total of 75 adult AML patients from July 2024 to June 2025 at the Fourth Affiliated Hospital of Guangxi Medical University and Affiliated Hospital of Hunan Normal University were enrolled. BM plasma levels were prospectively measured for IL-1β, IL-1RA, IL-2, IL-4, IL-6, IL-10, IL-12, IL-13, IL-15, IL-17, IL-18, IL-21, TNF-α, TGF-β and IFN-γ. This study was conducted in accordance with the Declaration of Helsinki and was approved by the Institutional Review Board at the Affiliated Hospital of Hunan Normal University. The diagnosis and classifications of the patients were based on the French-American-British (FAB) classification (Bennett et al., 1985) and 2022 WHO criteria (Khoury et al., 2022). FLT3 mutations in our study included both ITD and TKD mutations, all AML patients with NPM1 mutations diagnosis reached the requirement of blasts ≥20% (Khoury et al., 2022). Samples were collected from patients at different stages of AML, including patients with ND-AML (n = 41), CR (n = 19), and R/R-AML (n = 15). Relapsed and CR were defined according to the European LeukemiaNet (ELN) recommendations (Döhner et al., 2022). The R/R cohort include patients relapsed after chemotherapy-induced remission who did not undergo HSCT. BM plasma cytokines were assessed in ND-AML patients before initiating treatment, CR patients before consolidation chemotherapy, and R/R-AML patients before reinduction chemotherapy. All patients received standard induction chemotherapy according to the Chinese guidelines for the diagnosis and treatment of adult AML (Leukemia and Lymphoma Group, 2023; Chinese Society of HematologyChinese Medical Association and Chinese Medical Doctor Association, 2018), followed by consolidation chemotherapy or HSCT. The efficacy was evaluated as CR or ICR within one initial induction chemotherapy cycles. CR patients contains first CR, CR with partial hematologic recovery (CRh), and CR with incomplete hematologic recovery (CRi). ICR patients contains morphologic leukemia-free state (MLFS), partial remission, and non-response (NR). All patients were followed up to death or the research deadline.

### 2.2 Clinical variable stratification and characteristic analysis

Clinical laboratory variables were divided into tertiles (ND-AML, CR, and R/R-AML) based on the disease status. This allowed assessment of the relationship between cytokine distribution trends and disease status as well as treatment response. Differences across tertiles were assessed using the Kruskal–Wallis test, followed by pairwise Wilcoxon rank-sum tests for *post hoc* analysis. *P* values were adjusted for multiple comparisons using the Benjamini–Hochberg method.

### 2.3 Determination of BM plasma cytokines concentration

All BM samples, obtained at diagnosis (ND-AML), before the first consolidation chemotherapy (patients with complete remission, CR) and before the initiation of reinduction chemotherapy (R/R-AML), were collected in heparin-anticoagulant tubes. After collection, BM samples were centrifuged at 500 × g for 10 min. Then aliquots of plasma samples, were divided into portions and immediately stored at −80 °C until the ELISA was performed. All cytokines were analyzed using enzyme-linked immunosorbent assay (ELISA) kits (Ruixin Biotech, Quanzhou, China) according to the manufacturer’s manual. The concentrations of IL-1β, IL-1RA, IL-2, IL-4, IL-6, IL-10, IL-12, IL-13, IL-15, IL-17, IL-18, IL-21, TNF-α, TGF-β and IFN-γ were measured. The lower levels of assay sensitivity were shown in Supplementary Table S1.

### 2.4 Endpoints and statistical methods

CR was defined using standard criteria (e.g., <5% blasts in B aspirate, an absolute neutrophil count ≥1 × 10^9^/L, a platelet count ≥100 × 10^9^/L, and with no extramedullary leukemia) (Döhner et al., 2022). We were primarily interested in the association between cytokine levels and the disease state. To confirm that any relation between cytokine levels and disease state and the relation between cytokine levels and other covariates, multivariate analyses used the logistic regression for CR. Logistic regression was used to identify pretreatment covariates predictive of CR. Patients with missing data were excluded from the multivariate analyses.

**FIGURE 1:**
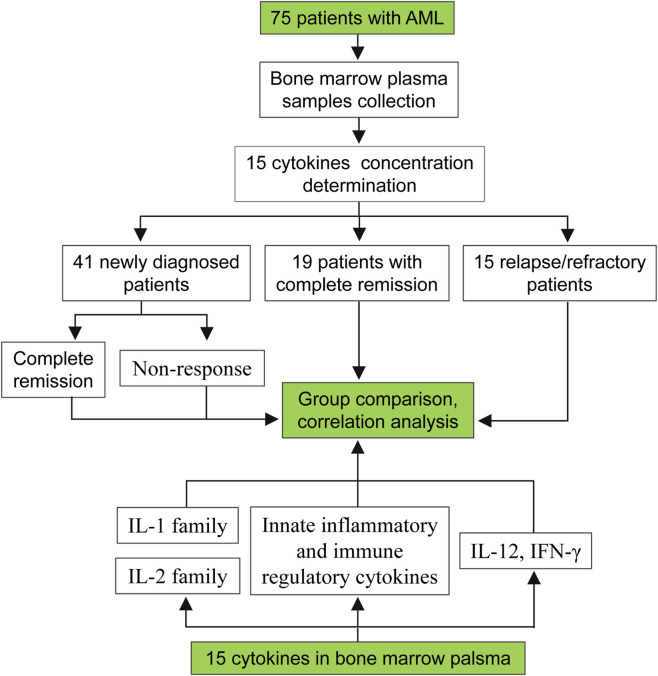
The experimental design and workflow of this study.

Descriptive statistics were reported as median (interquartile range, IQR) for continuous variables, and frequencies (percentages, %) for categorical variables. Pearson’s Chi-square test was used to compare categorical variables between groups. ANOVA or Kruskal–Wallis H-test was used to compare multiple groups, while t-test or Mann-Whitney U-test was used to compare two groups. For all tests, a *P*-value <0.05 indicated statistical significance. Analyses were performed using IBM SPSS Statistics 19.0 and GraphPad Prism 8.0. The overall technical workflow of this study is summarized in Figure 1.

## 3 Results

### 3.1 Clinical features of enrolled patients with AML at the time of diagnosis

Based on the disease status at the time of BM sample collection, patients with AML were divided into three groups (newly diagnosed, complete remission, relapsed/refractory), and their clinical parameters at initial diagnosis were compared. As shown in Supplementary Table 2, no significant differences in age, gender, white blood cell, hemoglobin, platelets, LDH, or BM blasts were found among these patient groups (*P* > 0.05). Genetic risk stratification was based on the 2022 ELN criteria (Döhner et al., 2022). Karyotypes classification was based on the 2022 WHO criteria (Khoury et al., 2022). There were no differences in ELN risk stratification, karyotypes classification, FLT3 mutation status and NMP1 mutation status among the three groups (*P* > 0.05). In summary, there was no significant difference in clinical characteristics among the three groups of patients at the initial diagnosis.

### 3.2 Cytokine analysis reveals quantitative alterations in the BM milieu during AML progression

Cytokines levels and their BM microenvironment composition were assessed in AML using ELISA. The concentrations of 15 cytokines were compared across different AML disease status (ND-AML, CR, and R/R-AML). The 15 cytokines were categorized into four groups (IL-1 family, IL-2 family, IL-12 and IFN-γ, and innate inflammatory and immune regulatory cytokines) (Kureshi and Dougan, 2025) for analysis (Figure 1).

**FIGURE 2:**
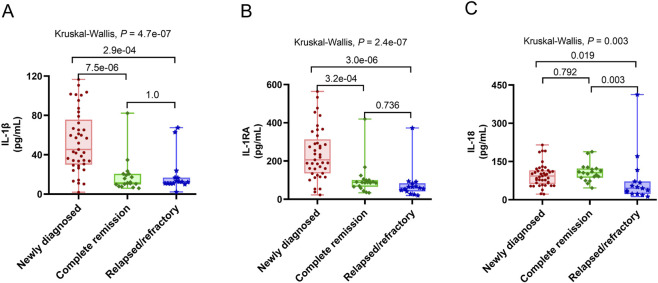
ELISA kits analysis of concentration of IL-1 family cytokines in BM plasma. Box plots show the statistically different distribution of IL-1β **(A)**, IL-1RA **(B)**, and IL-18 **(C)** between patients with newly diagnosed AML patients (ND-AML), complete remission patients (CR), and relapsed/refractory AML patients (R/R-AML).

We observed significant differences in the IL-1 family IL-1β and IL-1RA between the ND-AML and CR groups (*P* < 0.001), as well as differences between the ND-AML and R/R-AML groups (*P* < 0.001) (Figures 2A,B). The levels of IL-18 in patients with ND-AML and CR were significantly higher than those patients with R/R-AML (Figure 2C). The concentration of IL-1β and IL-1RA showed no difference between the CR and R/R-AML groups (*P* > 0.05, Figures 2A,B).

**FIGURE 3:**
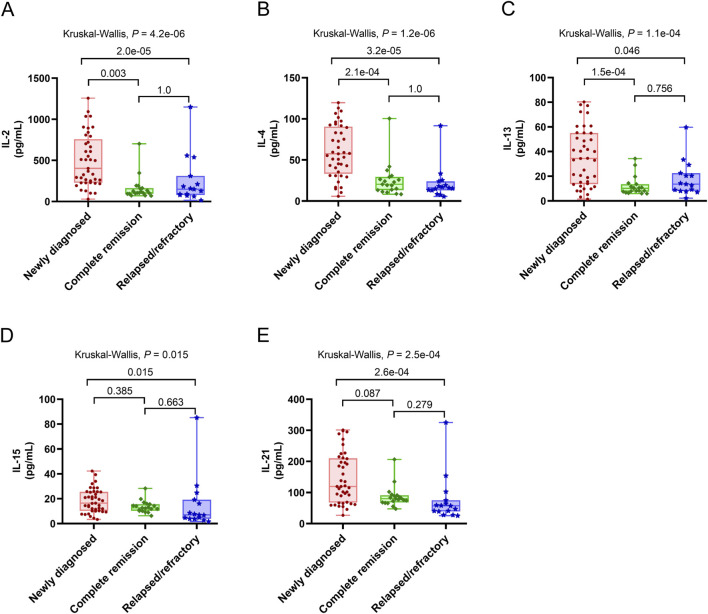
ELISA kits analysis of concentration of IL-2 family cytokines in BM plasma. Box plots show the statistically different distribution of IL-2 **(A)**, IL-4 **(B)**, IL-13 **(C)**, IL-15 **(D)**, and IL-21 **(E)** between patients with ND-AML, CR, and R/R-AML.

Notably, the concentration of IL-2 family, including IL-2, IL-4, and IL-13, in the ND-AML group was higher than in the CR and R/R-AML groups (*P* < 0.01, Figures 3A–C), while the levels of IL-15 and IL-21 decreased in the R/R-AML group compared to the ND group (*P* < 0.05) and their content showed no statistical difference between the ND-AML and CR groups, or between the R/R-AML and CR groups (*P* > 0.05) (Figures 3D,E).

**FIGURE 4:**
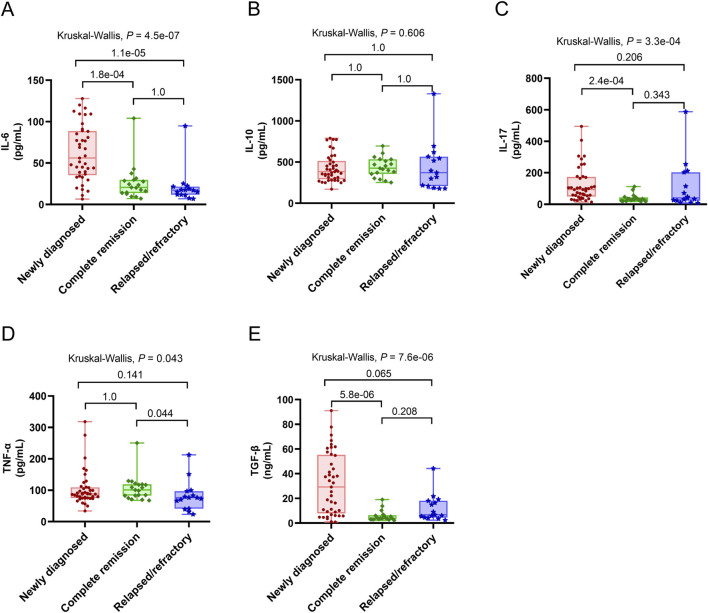
ELISA kits analysis of concentration of innate inflammatory and immune regulatory cytokines in BM plasma. Box plots show the statistically different distribution of IL-6 **(A)**, IL-10 **(B)**, IL-17 **(C)**, TNF-α **(D)**, and TGF-β **(E)** between patients with ND-AML, CR, and R/R-AML.

In innate inflammatory and immune regulatory cytokines, we observed a significant reduction in the IL-6 (median: 62.3 in ND-AML, and 25.9 in CR; *P* < 0.001, Figure 4A), IL-17 (median: 125.7 in ND-AML, and 38.7 in CR; *P* < 0.001, Figure 4C), TNF-α (median: 106.3 in CR, and 81.5 in R/R-AML, *P* < 0.05, Figure 4D) and TGF-β (median: 31.7 in ND-AML, and 5.7 in CR; *P* < 0.001, Figure 4E) across disease status. The concentration of IL-6 in the R/R-AML group also decreased compared to the ND group (*P* < 0.05, Figure 4A). In parallel, IL-6 (between CR and R/R-AML), IL-10 (across ND-AML, CR and R/R-AML), IL-17 (between ND-AML or CR and R/R-AML), TNF-α (between CR or R/R-AML and ND-AML), and TGF-β (between ND-AML or CR and R/R-AML) concentrations showed no statistical difference (*P* > 0.05, Figures 4A–E).

**FIGURE 5:**
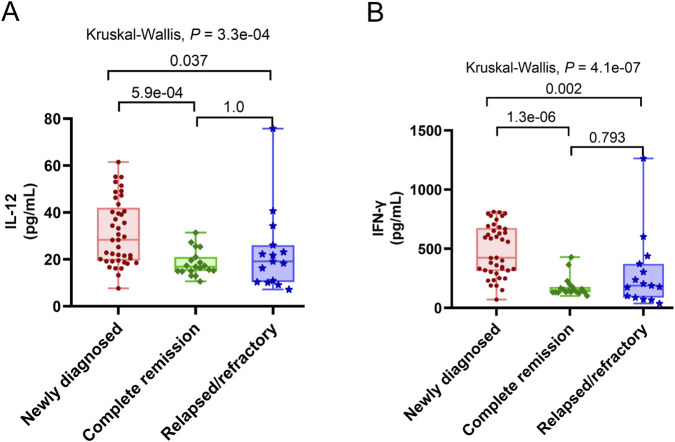
ELISA kits analysis of concentration of BM plasma cytokines IL-12 and interferons (IFN)-γ. Box plots show the statistically different distribution of IL-12 **(A)**, and IFN-γ **(B)** between patients with ND-AML, CR, and R/R-AML.

Additionally, IL-12 (median: 31.4 in ND-AML, and 18.3 in CR) and IFN-γ (median: 484.2 in ND-AML, and 175.6 in CR) levels were reduced in patients with ND-AML compared with those with CR (*P* < 0.001); as well as reduction in ND-AML group compared with the R/R-AML group (*P* < 0.05) (Figures 5A,B). However, no significant differences in the IL-12 and IFN-γ between patients with CR and those with R/R-AML were observed (Figures 5A,B).

### 3.3 BM plasma cytokine levels correlate with ELN risk and laboratory data

**FIGURE 6:**
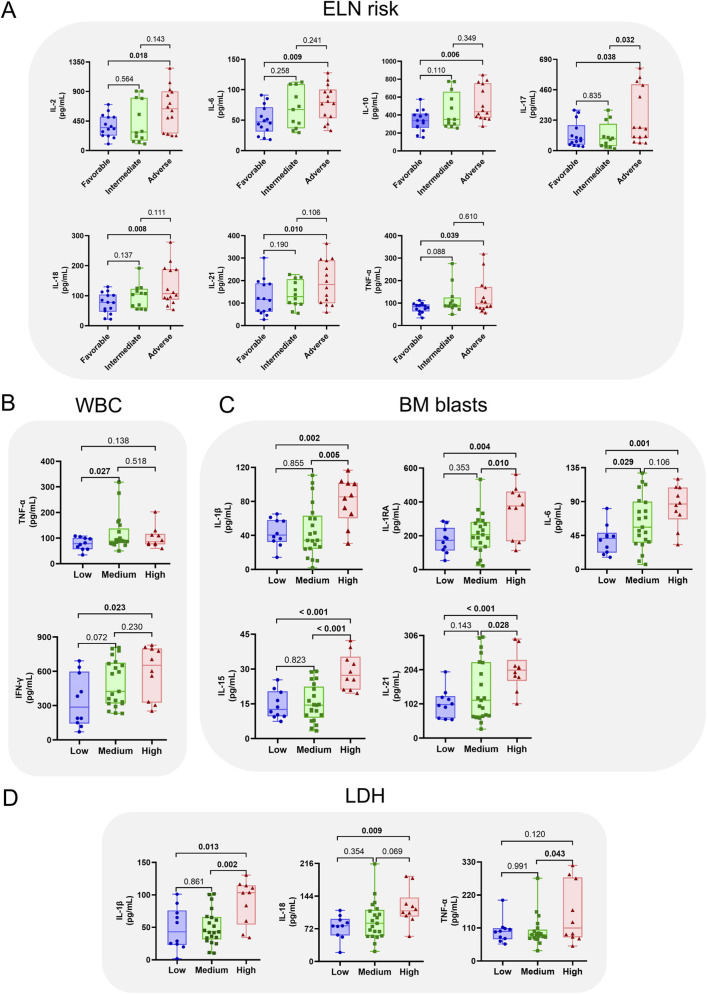
Identified BM plasma cytokines correlate with ELN risk and clinical laboratory data **(A)** Boxplots reveal statistically significant differences in the distribution of cytokines concentrations among different ELN risk groups. **(B-D)** Boxplots reveal statistically significant differences in the distribution of cytokines concentrations according to the levels of the following laboratory parameters: white blood cells (WBC) **(B)**, BM blats **(C)**, and LDH **(D)**.

Next, we evaluated the correlation between BM plasma cytokines and the ELN risk favorable, intermediate, and adverse in patients with AML, uncovering significant immunological and inflammatory differences linked to disease severity (Figure 6A). Specifically, IL-2, IL-6, IL-10, IL-17, IL-18, IL21, and TNF-α showed a significant increase in adverse than that in favorable groups (*P* < 0.05), with a marked elevation of IL-17 in adverse group compared with medium group (*P* = 0.032). Overall, these results indicate a possible trend of changes in cytokines composition within the BM microenvironment during disease progression, as reflected by differences observed in selected subsets.

Furthermore, we examined the correlation between BM plasma cytokines and various laboratory parameters, including white blood cells (WBC), BM blasts, and LDH. To evaluate immunological and inflammatory variation, clinical variables were stratified into three groups (low, medium, and high) based on the 25th and 75th percentiles of their distribution of measurement result range (Figures 6B–D). Among the 15 cytokines, medium WBC levels were associated with an increased concentration of TNF-α, whereas IFN-γ levels were raised in the medium (a trend of differentiation, *P* = 0.072) and high group (*P* < 0.05, Figure 6B). A comparable pattern was observed for BM blasts, with a increase in IL-1β, IL-1RA, IL-15 and IL-21 in the high group than that of low and medium groups. Similarly, the concentration of IL-6 increased with in the medium and high groups (*P* < 0.05, Figure 6C). Among the parameters, LDH levels were strongly associated with changes in IL-1 family composition, with IL-1β and IL-18 levels increased in the high-LDH group. Finally, TNF-α concentrations peaked in the high LDH group, with significant increase than that of the medium group (*P* < 0.05, Figure 6D).

### 3.4 High IL-12, IL-18, and IL-21 predict treatment response in patients with AML

We assessed the impact of BM plasma cytokines on treatment response among patients with AML (n = 41). Clinical characteristics and cytokines concentration between the CR and incomplete remission (ICR) groups were compared and presented in Supplementary Table 3. The results showed that the LDH levels of the ICR group were higher than that of the CR group [552 (316, 1666) vs. 372 (73, 756), *P* = 0.025]. The proportion of ELN risk-favorable AML patients in the ICR group was lower than that of the CR group [3 (15.8%) vs. 11 (50.0%), *P* = 0.045]. However, we did not observe significant differences in age, gender, and BM blasts between CR and ICR groups (*P* > 0.05). Together, these results indicate that the lower LDH levels and ELN risk-favorable represent better AML treatment response.

Notebaly, higher levels of IL-12 were found to be associated with better treatment response (*P* = 0.006). Remarkable differences were also observed in the distributions of IL-18 and IL-21. As shown in Supplementary Table 3, The IL-18 levels of the CR group were higher than that of the ICR group [104.7 (78.0, 134.8) vs. 69.3 (53.7, 103.8), *P* = 0.005], and the IL-21 concentrations of the CR group were also increased compared to the ICR group [183.3 (96.0, 218.1) vs. 101.9 (59.9, 159.6), *P* = 0.034]. However, there was no difference in other BM plasma cytokines between the CR and ICR groups. To sum up, these results suggested that high levels of BM plasma IL-12, IL-18, and IL-21 were associated with improved treatment response and may serve as valuable biomarkers for predicting treatment response in AML.

## 4 Discussion

Cytokines exert profound influences on the progression of hematopoietic malignancies such as AML (Binder et al., 2018). Critical roles of cytokines in the context of immunity and inflammation have obtained great attention. While anti-inflammatory mediators such as IL-10 and TGF-β tend to hinder the progression of AML, pro-inflammatory mediators such as IL-1 β, IL-6, and TNF-α appear to promote AML proliferation and aggressiveness (Karimdadi Sariani et al., 2021; Allen et al., 2025). Dysregulation of the interactions between anti- and pro-inflammatory cytokines in AML may create a pro-tumorigenic microenvironment affecting AML cell growth, progression and drug-resistance (Binder et al., 2018; Luciano et al., 2022; Wang et al., 2023; Hou et al., 2024). Thus, this study aimed to map alterations in BM plasma cytokines composition among the AML progression and treatment response. Furthermore, we correlated BM immune and inflammatory cytokines composition with clinical characteristics to identify biomarkers associated with improved response to therapy in patients with AML.

Leukemic cells are dependent on the microenvironment of the host. The factors responsible for the survival of leukemic cells have not been fully identified to date (Kováč et al., 2014). As most of the attention has been paid to the biology of the leukemic cells themselves, the importance of soluble factors produced by leukemic cells or by their surroundings is largely unclear. Data showing a suppressive effect of adult AML patient BM plasma on the growth of hematopoietic progenitor cells have been reported. This effect was abolished by anti-TNF alpha and anti-adiponectin antibodies (Iversen and Wiig, 2005). Published studies have identified that IL-12, IL-15, and IL-18-induced memory-like NK cells elicit enhanced anti AML (Romee et al., 2016; Terrén et al., 2022; Boieri et al., 2017). Our data showed that IL-12, IL-15, and IL-18 levels were significantly decreased in R/R-AML compared to ND-AML patients, and IL-18 concentrations were lower in R/R-AML than that of CR groups. These results suggest that downregulation of these cytokines in BM microenvironment of R/R-AML patients maybe related to a abatement of the potential ability to induce memory-like NK cells triggering anti-leukemia effects (Romee et al., 2016). Thus, we speculated that ND-AML patients with high IL-12, IL-15, and IL-18 maintains compensatory ability to trigger against leukemia effects (Romee et al., 2016), and the CR patients’ BM may retain a certain ability to stimulate anti-leukemia.

Pro-inflammatory IL-6 and TNF-α in peripheral blood plasma have been reported to play a critical role in the leukemic blast formation and growth (Carey et al., 2017; Karimdadi Sariani et al., 2021). Similarly, our results showed that the ND-AML patients’ BM plasma IL-6 levels were higher than that of CR and R/R-AML patients, indicting a important role in initial AML. While TNF-α maintained relatively high levels in BM of ND-AML (a trend of difference, *P* = 0.141) and CR (*P* < 0.05) patients compared with R/R-AML. Horacek et al. evaluated the serum levels of 17 cytokines in AML patients at diagnosis and 6 months after the last chemotherapy completion with durable CR. They found that IL-17, vascular endothelial growth factor (VEGF), and epidermal growth factor (EGF) reflecting the disease activity or alterations caused by leukemia treatment (Horacek et al., 2013). Recent study reported that AML stem cells remodeled the BM niche *via* TGF-β, creating a self-sustaining environment. Consistently, we found that IL-17 and TGF-β concentrations in BM plasma were high at newly diagnosed, decreased after complete remission, and increased (a trend of difference, *P* = 0.343 and 0.208 respectively) again at relapse, reflecting the disease activity. Serum IL-21 at diagnosis is identified as favourable outcome and higher CR rates in AML patients that underwent high-dose chemotherapy (Rubino et al., 2024). Rubino et al. have identified CD4^+^ T cell-derived IL-21 as an important negative regulator of self-renewal of leukemia stem cells (LSCs) and targeting IL-21/IL-21R signaling on LSCs may be an approach to treat AML (Rubino et al., 2024). Our study showed that IL-21 in BM was markedly decreased in R/R-AML compared with ND-AML, suggesting that the attenuation of IL-21 in AML patients may be beneficial for leukemia recurrence or refractory. It is worth noting that both IL-1 family cytokines IL-1β and IL-1RA, and IL-2 family members IL-2, IL-4 and IL-13 were increased ND-AML than that of CR and R/R-AML groups. The differences in these cytokine composition in BM plasma may be reported for the first time, however, the significance deserves further exploration.

Through comparing BM plasma cytokines and clinical characteristics at diagnosis between CR and ICR groups in newly diagnosed AML patients undergoing first induction chemotherapy cycle, we observed significant differences. The BM concentrations of cytokines IL-12 and IL-18, which were identified as triggering anti-leukemia (Romee et al., 2016; Boieri et al., 2017), and cytokine IL-21, which was reported as inhibiting self-renewal of LSCs (Rubino et al., 2024), were significantly higher in CR than that of ICR groups. These results suggested that higher IL-12, IL-18, and IL-21 in BM plasma were related to better treatment outcomes or higher CR rates. In addition, we observed that low LDH levels and ELN risk-favorable were associated with better treatment response. Our study is the first to comprehensively evaluate the changes in BM plasma cytokines subsets in AML before and after induction chemotherapy. As reported, the recovery of several cytokines after treatment is associated with disease status, and the immune and inflammatory recovery of patients after treatment is heterogeneous. The heterogeneity may explain why cytokines composition in the different disease state showed correlation with AML prognosis in this study. The differences between the CR and ICR groups also suggest that specific cytokines at ND-AML status may be used to predict AML treatment response, highlighting the necessity of dynamic monitoring in BM plasma.

## 5 Conclusion

Based on the changes in cytokines across different disease status, we analyzed the correlation among cytokines levels, treatment response, and progression in AML patients. Our study indicates a sight of BM microenvironment heterogeneity in immune and advanced disease stages. However, it is obliged to emphasize that a limitation in our study lies in the lack of a cohort that includes healthy controls, the relatively small sample size of the patients cohort, and the lack of longitudinal paired samples for all patients. Indeed, previous studies have shown that the concentrations of cytokines in blood and BM of healthy hematopoietic stem cell donors were at an extremely low level, suggesting that the alterations of BM cytokines were more likely to be observed in different disease status. Collectively, these findings suggest that distinct levels of the BM cytokines predict leukemia progression and treatment response. And this is the first report on the association between BM plasma cytokines at diagnosis of AML and the disease status. Further analyses are required to explore mechanisms implicated in the deregulation of these cytokines.

## Data Availability

The original contributions presented in the study are included in the article/Supplementary Material, further inquiries can be directed to the corresponding author.
